# Chronic disease prevention policy in British Columbia and Ontario in light of public health renewal: a comparative policy analysis

**DOI:** 10.1186/1471-2458-13-934

**Published:** 2013-10-08

**Authors:** Anita Kothari, Dana Gore, Marjorie MacDonald, Gayle Bursey, Diane Allan, Jennifer Scarr

**Affiliations:** 1 Faculty of Health Sciences, University of Western Ontario, Ontario N6A 5B9, Canada; 2 School of Nursing, University of Victoria, British Columbia V8P 5C2, Canada; 3 Chronic Disease and Injury Prevention, Peel Public Health, Ontario L5M 2C1, Canada; 4 Vancouver Coastal Health, British Columbia V5Z 4C2, Canada

**Keywords:** Health policy analysis, Public health, Health promotion, Healthy eating, Physical activity, Framework for Core Functions in Public Health, Ontario Public Health Standards, Public health systems

## Abstract

**Background:**

Public health strategies that focus on legislative and policy change involving chronic disease risk factors such as unhealthy diet and physical inactivity have the potential to prevent chronic diseases and improve quality of life as a whole. However, many public health policies introduced as part of public health reform have not yet been analyzed, such as in British Columbia and Ontario. The purpose of this paper is to present the results of a descriptive, comparative analysis of public health policies related to the Healthy Living Core Program in British Columbia and Chronic Disease Prevention Standard in Ontario that are intended to prevent a range of chronic diseases by promoting healthy eating and physical activity, among other things.

**Methods:**

Policy documents were found through Internet search engines and Ministry websites, at the guidance of policy experts. These included government documents as well as documents from non-governmental organizations that were implementing policies and programs at a provincial level. Documents (n = 31) were then analysed using thematic content analysis to classify, describe and compare policies in a systematic fashion, using the software NVivo.

**Results:**

Three main categories emerged from the analysis of documents: 1) goals for chronic disease prevention in British Columbia and Ontario, 2) components of chronic disease prevention policies, and 3) expected outputs of chronic disease prevention interventions. Although there were many similarities between the two provinces, they differed somewhat in terms of their approach to issues such as evidence, equity, and policy components. Some expected outputs were adoption of healthy behaviours, use of information, healthy environments and increased public awareness.

**Conclusions:**

The two provincial policies present different approaches to support the implementation of related programs. Differences may be related to contextual factors such as program delivery structures and different philosophical approaches underlying the two frameworks. These differences and possible explanations for them are important to understand because they serve to contextualize the differences in health outcomes across the two provinces that might eventually be observed. This analysis informs future public health policy directions as the two provinces can learn from each other.

## Background

Chronic diseases, defined by the World Health Organization as “diseases of long duration and generally slow progression”, are becoming a serious problem worldwide [1]. Some major categories of chronic disease that have high morbidity and mortality rates around the world include cardiovascular diseases, cancer, chronic respiratory diseases and diabetes.

WHO estimated that chronic diseases accounted for 46% of the global burden of disease in 2001; this proportion is expected to climb to 57% by 2020 [2]. Additionally, as the global proportion of deaths from infectious diseases has decreased, the proportion attributed to chronic diseases has increased. Chronic diseases contributed to 63% of deaths around the world in 2008 and are projected to contribute to 75% of deaths by the next decade [2,3]. This epidemiologic shift is occurring in both developed and developing countries, and Canada is no exception. Forty-two per cent of Canadians over the age of 12 are living with a chronic condition (either a chronic disease or a risk factor for a chronic disease), a figure that grows progressively larger for older age groups [4]. The majority of chronic disease-related deaths are attributed to cardiovascular diseases (CVD), though conditions such as cerebrovascular diseases, diabetes, respiratory illnesses, and cancers are common as well [2].

The causes of chronic diseases are complex and rooted in social and economic structures, tied to social determinants of health such as income inequality, education, working conditions, food insecurity and housing. However, the WHO has made it clear that it is also possible to prevent some chronic diseases through the reduction of key risk factors. The four main behavioural risk factors are unhealthy diet, physical inactivity, tobacco use and the harmful use of alcohol [3]. Public health strategies such as policy changes are key to preventing chronic diseases, particularly at the population level. Some proven and/or promising strategies include legislation to protect people from tobacco smoke, high taxes on tobacco, and policies to reduce salt content in food and replace trans-fats with polyunsaturated fat [3]. These targeted policy-level solutions are crucial to address the social determinants of health, the unequal distribution of which systematically sustain risk behaviours related to diet, nutrition, tobacco use, and alcohol consumption [5].

Strategies that focus on legislative and policy change have the potential to alter the lives of Canadians in fundamental ways that not only prevent chronic diseases, but also improve quality of life as a whole. However, many public health policies introduced as part of public health reform have not yet been analyzed, individually or for their potential synergistic effect, because they are still in the initial stages of implementation. This is the case in two Canadian provinces currently undertaking a process of public health system reform in which a major public health policy intervention is being implemented in each province. In British Columbia (BC), the Framework for Core Functions in Public Health [6], and in Ontario (ON) the new Public Health Standards [7] laid out policy frameworks for implementing core public health programs for population health assessment; health surveillance; disease and injury prevention; health promotion and health protection [8]. Aspects of the policy related to chronic disease prevention were selected for closer examination in this paper (and the larger research program) as one exemplar to illustrate the implementation of the larger frameworks.

The public health systems in ON and BC are organized very differently (see Additional file 1), and each of these provincial policies takes a somewhat different approach to chronic disease prevention. These naturally occurring variations provide a unique opportunity for a natural experiment of sorts to study the implementation of public health renewal in the two provinces. ON and BC were selected for analysis because, in addition to undergoing simultaneous public health reform processes, these two provinces are quite distinct in terms of geography, population demographics, economics, organizational structures and governance. The analysis described in this paper reports on a first step in a larger study (Renewal of Public Health Systems – RePHS) that explores the implementation and impact of these public health policy interventions in BC and ON, with particular respect to: chronic disease prevention/healthy living and sexually transmitted infection prevention (see http://www.uvic.ca/research/groups/cphfri/projects/currentprojects/rephs/index.php). Our purpose in this paper is to present the results of a descriptive, comparative analysis of public health policies related to the Healthy Living (HL) Core Program in BC and Chronic Disease Prevention (CDP) Standard in ON that are intended to prevent a range of chronic diseases by promoting healthy eating and physical activity.

Tobacco prevention and control were excluded from this analysis for several reasons: a) their unique long and rich history in both provinces, b) the fact that related legislative and policy measures were more restrictive and focused on risk-mitigating behaviours (e.g. tobacco cessation) and protecting the public from second-hand smoke, rather than health-promoting behaviours (e.g. healthy eating) [9], and c) an analysis of tobacco policy will be reported in a separate paper.

Not only will this analysis illustrate the variations in CDP policy in two provinces but will also provide a baseline against which to judge implementation changes in policy over time. Evaluating the outcomes of policy interventions can be particularly challenging without baseline data, and it is difficult to generate evidence to assess the whether the policy has been successful [10]. As the RePHS project moves forward, further developments in policy documents, program implementation and impacts will be assessed against this baseline analysis.

*The Canadian Context*: Canada is a country composed of 10 provinces and 3 northern territories. Although on a national level Canada’s health-care system is publically funded and guided by the Canada Health Act, jurisdiction over the majority of health care services, funding structure and delivery lies with the provinces/territories. Residents are required to pay into mandatory provincial insurance plans, which then allow them to receive medically necessary physician and hospital services at no cost up front, while health care professionals bill the provincial insurance provider. Provincial plans may cover other services (for example dental care, eye care and drugs), but they are not required to do so, and in many cases private insurance plans supplement provincial insurance to fill this gap. Public health in most provinces in Canada is integrated into regional health authorities that are responsible for providing all health care services. The exception is Ontario in which public health does not fall under the health care system per se. It is its own separate, albeit linked, system (see Additional file 1). However, as noted above, public health services are not funded by federal government transfer payments as are other health care services.

Public health is defined by the Public Health Agency of Canada Act as “population-focused and includ[ing] disease surveillance, disease and injury prevention, health protection, health emergency preparedness and response, health promotion, and relevant research undertakings” [11]. There is shared responsibility and jurisdiction over public health between the federal government and the provinces/territories. While protecting Canadians from the spread of diseases (e.g. quarantine) falls within national jurisdiction, the provinces and territories structure, fund, and deliver public health services [11].

*Federal Role*: National public health reform became a priority in Canada when public health emergencies such as SARS revealed serious gaps and failings in the existing public health system. In 2004/2005, the federal government provided $400 million in funds specifically to enhance public health capacity, including in the area of chronic disease prevention [6]. While a large portion of these funds went to the provinces for public health revitalization and established important structures such as the Ontario Agency for Health Protection and Promotion (now known as Public Health Ontario), there were also important developments on the national stage. The resulting establishment of PHAC, the Federal/Provincial/Territorial Health Promotion Expert Committee, and six national public health collaborating centres around the country focused on health promotion issues have significantly impacted research, knowledge and programming around chronic diseases [12]. The federal department of health, Health Canada, is also involved in developing policy, promoting research, and funding projects related to health promotion and chronic disease prevention. Some notable initiatives with federal involvement include the Canadian Heart Health Initiative, the Canadian Diabetes Strategy, the Canadian Cardiovascular Disease Action Plan, and ParticipACTION, which demonstrate a policy and program environment that is favourable to chronic disease prevention strategies [12,13]. It should be noted that the extent to which the federal government is involved with chronic disease prevention is debatable. Although it is purportedly committed to health surveillance, high-level policy setting and national leadership, the majority of health promotion and chronic disease prevention programming falls to the provinces [11]. For more information around federal and provincial organizations and structures that impact chronic disease prevention policy and programming, please see Additional file 1.

### The policy interventions

#### BC

**Figure 1:**
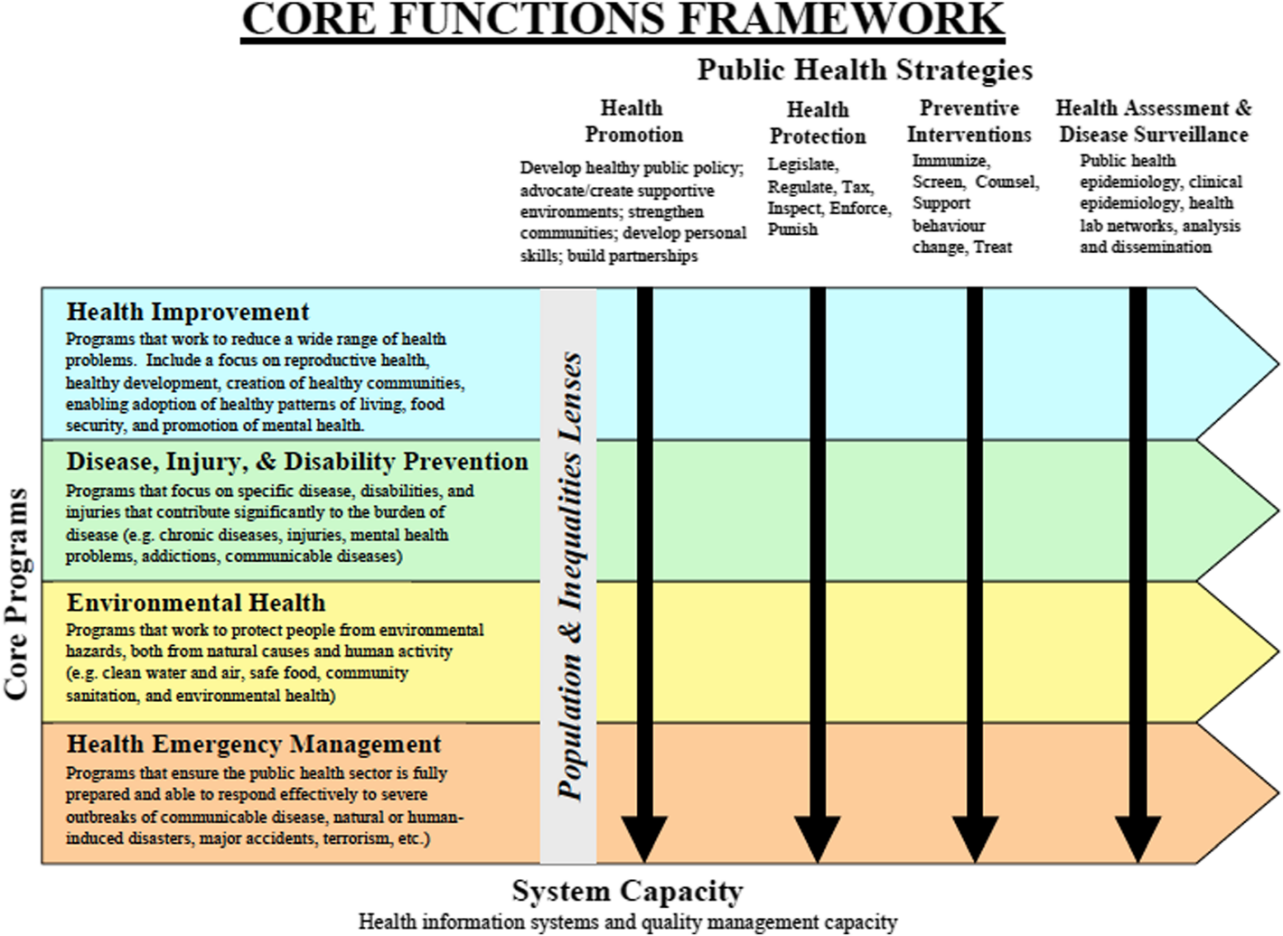
**BC Core Functions of Public Health Framework.** This framework outlines the core program categories, the main public health strategies used in the implementation of the programs, the population and inequity lenses that are applied to all programs, and system capacities necessary to carry them out.

The original BC Core Functions of Public Health Framework (CF Framework) that was created in 2005 [14] identified twenty-one core public health programs that encompass the areas of health improvement; disease, disability and injury prevention; environmental health; and health emergency management (see Figure 1). There was a staggered implementation of these core programs by health authorities (HA) beginning in 2007 and continuing until 2010. Although each core public health program in the BC framework was defined originally as an individual and distinct program, most HAs clustered their core programs in some way, often by population, to achieve some economies of scale and to take advantage of synergies in delivery and implementation. HAs are responsible for the implementation and quality of their programs; they are expected to develop programs/services and a performance improvement plan for each core program in line with findings of a provincial evidence review and a model core program paper, a logic model, outcome indicators and regional needs.

The public health strategies employed to deliver core programs include health promotion, health protection, prevention, and health assessment/disease surveillance. A population and an equity lens were expected to be applied to all programs and strategies to address health inequities and to ensure that the needs of specific populations were met, including but not restricted to vulnerable or marginalized groups. Finally, the framework included a Core Public Health Capacity component that identified the health information systems, quality management, research and knowledge development, and staff training and capacity development needed to apply public health strategies and implement core programs.

#### ON

**Figure 2:**
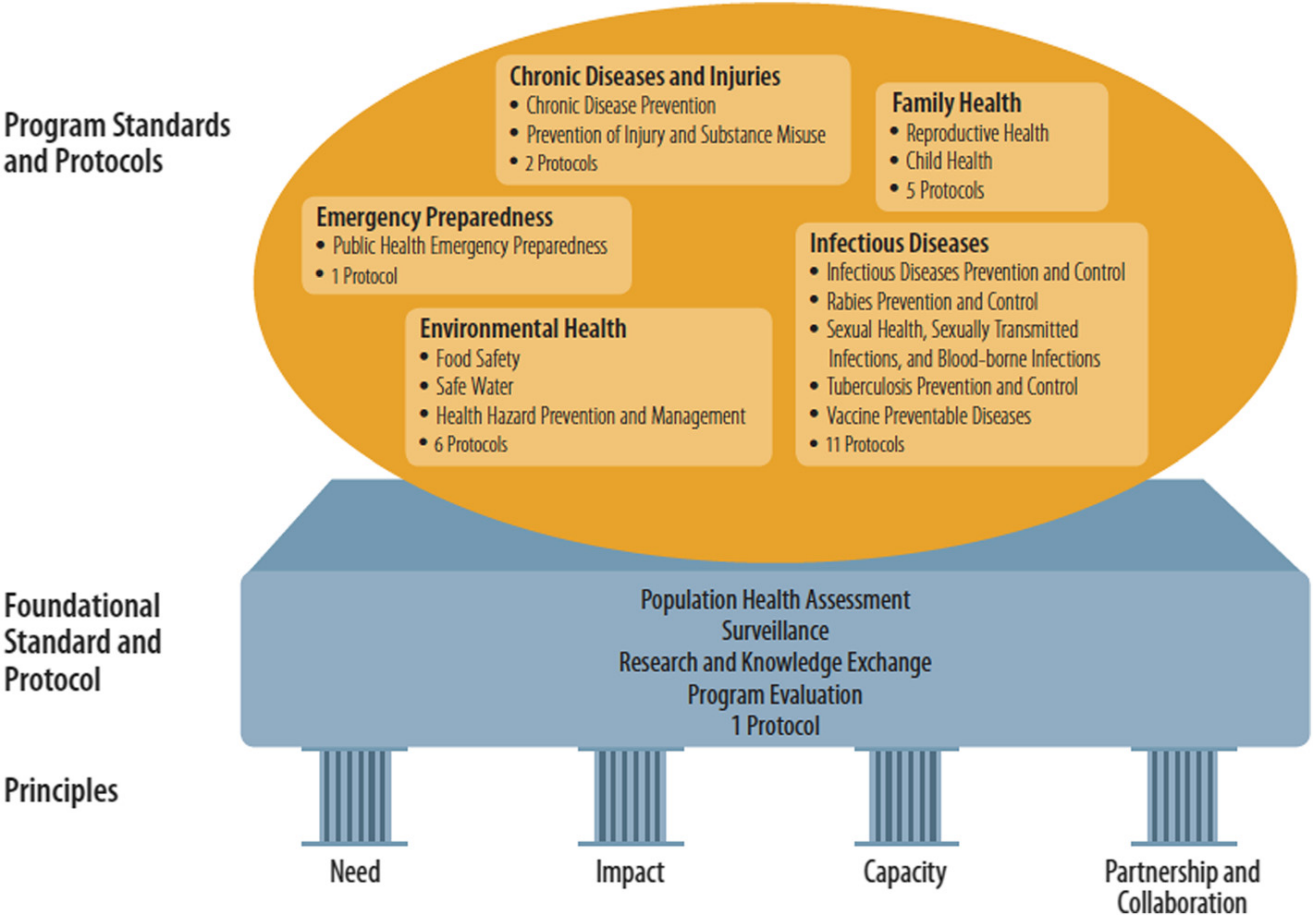
**Ontario Public Health Standards.** This framework demonstrates elements of the foundational standard and protocol that are used to implement the program standards and protocols (shown in the orange circle). The foundational standard in turn is supported by four principles that are represented by pillars.

The Ontario Public Health Standards outlined thirteen program standards in five areas: chronic diseases and injuries, family health, infectious diseases, environmental health and emergency preparedness (see Figure 2). All programs were based on a foundational standard that emphasized the importance of population health assessment, surveillance, research and knowledge exchange, and program evaluation. This foundational standard paralleled, in some ways, the Public Health Capacity component of the BC framework. While the MOHLTC was responsible for the majority of most standards, including the Foundational Standard, (during the period of this study) the Chronic Disease Prevention standard was directed by the former Ministry of Health Promotion (now absorbed into the Ministry of Health and Long-Term Care), and then administered by public health units [15]. The standard had specific goals, desired health and societal outcomes, and requirements attached to it that related to how the Board of Health in each unit would plan and deliver programming around assessment and surveillance, health promotion, policy development, and health protection [15]. There were also more detailed protocols and agreements for provincially funded programs that guided how Boards were meant to implement programming, such as the Nutritious Food Basket Protocol, the Population Health Assessment and Surveillance Protocol and Smokefree Ontario Service Agreements [15].

It should be noted that in ON, the Chronic Disease Prevention Standard encompassed physical activity and healthy eating. In BC, the policy Framework included two main core programs that address chronic disease prevention activities. The Healthy Living core program included physical activity and healthy eating while the Chronic Disease Prevention (CDP) core program included prevention activities for specific chronic diseases and may not be directly related to these areas, although the expectation was that by addressing physical activity and healthy eating, there will be prevention benefits for several of the chronic diseases that are the focus of the CDP core program (e.g., musculoskeletal disorders, respiratory diseases, and various forms of cancer). For the purpose of this paper we focus on the HL core program in BC and the CDP Standard in ON as they both include physical activity and healthy eating.

## Methods

### Study design

This study was designed as a descriptive comparative analysis of CDP policies in ON and BC using thematic content analysis of key policy documents. Health policy in this case is defined as “courses of action (and inaction) that affect the set of institutions, organizations, services and funding arrangements of the health system” [16], p.6. The research questions were: how do the recent CDP/HL policies compare across BC and ON in terms of their scope and focus, and what are some contextual explanations for these differences? What are the implications of these differences?

### Document search and selection strategy

The search for documents was done in two phases. In the first phase, in 2010, the research team performed a search, using different combinations of the words “healthy eating”, “active living”, “chronic disease prevention”, “policy”, “strategy” and “public health” for BC and ON documents using the Internet search engine Google and websites of relevant Ministries in both provinces (the former Ontario Ministry of Health Promotion, the Ontario Ministry of Health and Long-Term Care, the former BC Ministry of Healthy Living and Sport, and the BC Ministry of Health). No other search engines were used because all documents were available in the public domain. The second phase, in 2011, was conducted to ensure that the team had a comprehensive list of documents. In the second phase, in 2011, the research team was expanded to include key policy makers from each province. These individuals provided further guidance on additional government policy documents that would be useful to include in the analysis. The selection criteria also broadened to include documents from key non-governmental organizations (NGOs) that were implementing CDP/HL policies and programs at a provincial level (e.g., the BC Healthy Living Alliance).

Documents for those policies that operate at the provincial level were selected for analysis, with a publication date cut-off of September, 2010. Changes in provincial policy structures, which may be found in documents published after September, 2010, are not reflected in this analysis but will be examined in subsequent follow up analyses. Because the focus of this study was on provincial policy, only documents that identified CDP/HL policies at the provincial level were included; documents that addressed policies and initiatives at the regional health authority (in BC) and local health unit level (in ON) were not used for analysis but will be considered in other analyses being conducted for the larger RePHS study. The larger program of research will also go beyond policy to analyze the implementation and impact of public health programming in both provinces, including the impact of the different organizational, delivery and funding structures for public health in the two provinces. For example, it will examine to what extent in ON local government priorities affect public health programs, given that local governments fund a portion of public health activities.

Documents that provide an overview of public health reform at a national level were used for understanding context but not included in the document analysis. The final list of documents included in the analysis can be found in Table 1. They are categorized into core, supporting and contextual documents. Core documents form the heart of CDP/HL policy as envisioned by BC and ON governments. Beyond the core programs other government documents that related to these policies were seen as supporting documents, and contextual documents have non-governmental authors that provide a more complete picture of the formation and implementation of healthy living policies.

**Table 1:** List of Analyzed Documents for BC and ON

**BC**	**ON**
***Core documents***	***Core documents***
• A framework for core functions in public health: resource document [6]	• Ontario Public Health Standards (OPHS) [7]
	• OPHS: Requirements* [17]
• Public Health Renewal in British Columbia: An overview of core functions in public health [14]	• OPHS: Board of Health Outcomes *[18]
	• OPHS: Goals* [19]
• Model Core Program Paper: Healthy Living [20]	• OPHS: Societal Outcomes* [21]
• Model Core Program Paper for Prevention of Chronic Diseases [22]	• Comparison of 2008 OPHS and 1997 MHPSG* [23]
***Supporting***	• The nutritious food basket guidance document* [24]
• A framework for a provincial chronic disease prevention initiative* [25]	• Healthy Eating, Physical Activity and Healthy Weights: guidance document* [26]
• Delivering Effective, Integrated System of Primary and Community Care* [27]	• Population Health Assessment and Surveillance Protocol* [28]
	• Nutritious food basket protocol* [29]
***Contextual***	• OPHS: Chronic Disease Prevention Logic Model* [30]
• Implementation of healthy living as a core program in public health: final report* [31]	***Supporting***
• Leading British Columbia towards a healthy future* [32]	• Ontario’s action plan for healthy eating and active living* [33]
• An environmental scan on primary care and public health in the province of British Columbia* [34]	***Contextual***
	• From Vision to Action: A plan for the Ontario Agency for Health Protection and Promotion* [35]
	• Moving the Healthy Eating and Active Living Strategy forward in Ontario* [36]
	• Obesity: an overview of the current landscape and prevention-related activities in Ontario* [37]
	• Informing directions for chronic disease prevention and management in Ontario* [38]

*Policy documents that were identified in Phase 2 of the document search and selection strategy, as opposed to Phase 1.

### Document analysis

Documents (n = 31) were analysed using thematic content analysis [39] to classify, describe and compare CDP/HL policies in a systematic fashion. The analysts (AK, DG, MM, DA, GB), who were a mix of researchers and policy makers, initially read through the assigned documents to become familiar with the data. The first author created a preliminary coding framework with a codebook and operational definitions using a key policy document from each province; the framework reflected interest in how the policy tools were constructed, what they might achieve and any evaluation component [40]. The analysts then came together to discuss the framework until a common understanding of the definitions and analytical process was established. A detailed coding protocol was developed and taught to all coders using a short policy document as an example to ensure rigour and inter-coder reliability. Each document was then re-read and coded using the software NVIVO 9 to select sections of text and assign them to an *a priori* code in the framework. Data that did not fit any of the existing codes were assigned a new code. Only two new codes were created that did not exist in the preliminary framework - “motivators” and “barriers” were added to the category “contextual factors” as a way to capture aspects of the provincial context that acted as facilitators or barriers to CDP/HL policy. Codes were then compared within each document to create higher order categories with shared common characteristics and then the categories were compared across documents to identify broader themes [41]. Repeated team de-briefings were held to question the analysts’ assumptions about the data, and to ensure that the themes were reasonable and plausible. A summary of the results for each code was compiled by DG and discussed by all team members to interpret the findings. Topics most discussed during the team debriefings included the scope and breadth of CDP/HL policies in BC and ON and their respective policy documents. The policy maker team members were able to provide insights into policy legacies and how they shaped the new provincial standards and frameworks.

## Results

Three main categories emerged from the analysis of documents: 1) goals for CDP/HL in BC and ON, 2) components of CDP/HL, and 3) expected outputs of CDP/HL interventions. In the section below we describe these aspects of CDP/HL policy for BC and ON.

### BC

#### Goals for the healthy living core program

The overarching goal for the BC Healthy Living Program was “to optimize health by increasing the adoption of healthy behaviours by British Columbians” [20], p.10, whereas the goal for the BC CDP core program was “to improve the health and well being of British Columbians by preventing and/or reducing the incidence and prevalence of chronic disease among the population” [22], p.20. BC’s specific goals focussed on protective factors such as a positive psychosocial environment and community empowerment, which provided a balance between a strengths-based and a risks-based approach.

The approach to achieve these goals focused on tackling upstream risk factors, and used an evidence-based population health approach to achieve this. For example, one of BC’s priorities was that policies and practices were evidence-based. Evidence related to chronic diseases and strategies for their prevention was drawn from a variety of sources, both formal and experiential, e.g., studies, surveillance statistics, papers on best practices and policy directions, and past experience with other initiatives. The evidence was synthesized in a published formal evidence review that was readily available to the HAs and practitioners [42]. The importance of evidence is also found in the “System Capacity” component of the Core Functions Framework, which stressed that health information systems and research and knowledge development were necessary to implement all core programs.

BC explicitly employed an equity lens as well as a population lens in its public health framework to design appropriate interventions for specific populations. The equity lens is meant to be a perspective which is sensitive to the socioeconomic, political and cultural contexts that create health inequities, and so can be used to identify and track inequitable differences in health status. An equity lens lends itself to working to reduce inequities in health through analysis, community action and advocacy, while the population lens directs attention to particular groups, including but not restricted to vulnerable or marginalized groups. These two lenses were seen as distinct and each was meant to be applied to every intervention or core program. It was recognized in the core BC documents that interventions which were both universal and targeted towards vulnerable groups were necessary to ensure access and maximize equity.

#### Components of healthy living

BC documents listed four explicit components of healthy eating and active living strategies (see Table 2). Two components were advocating for public policy change and increasing awareness of the public and health professionals using social marketing and education. BC also included the community as an integral component, in the context of capacity building (Healthy Communities was another core public health program in BC that addressed the creation of healthy environments and settings for communities [43]). Capacity building is meant to empower individuals and communities to participate in and create sustainable changes to healthy living. For the fourth main component, BC stated the importance of programs and services that involve collaboration between different sectors such as HAs, community organizations and other partners.

**Table 2:** Explicit components of healthy living core program (BC) and chronic disease prevention standards (ON) CDP programming

	**BC [**[6]**], p. iii-iv**	**ON [**[33]**], p. 9**
**Explicit Components of Healthy Eating and Active Living Strategies**	**Advocacy and Public Policy**	**Champion Public Policy**
	**-** Influence policy at the community, regional, provincial and/or federal levels	-*Build partnerships* for [policy] change
Text is quoted from documents except for text in square brackets, which has been added by the authors for clarification. Certain text has also be italicized by the authors to emphasize areas of contrast between the two provinces.	**Public Education, Awareness and Social Marketing**	-Foster *learning and innovation* [with respect to collaborative action]
		-Invest in [program and policy] results
		**Promote Public Awareness and Engagement**
	- Provide educational materials, events and social marketing campaigns targeted to health professionals and *priority populations.*	-Support public education and marketing campaigns
		-Align public awareness efforts
		-Inform *parents, caregivers* and professionals
	**Community Capacity Building**	**Build Healthy Communities**
	**-***Enhance the community’s skills and ability to support healthy eating and physical activity*	-*Partner with Aboriginal communities*
		-*Promote healthy urban design*
		-*Help Ontarians access dietitians*
	***Programs and Services (Interventions)***	***Grow Healthy Children and Youth***
	**-***Interventions may be provided directly by the health authorities or, indirectly through collaboration with partners and community organizations. Intersectoral collaboration is integral to the CF Framework.*	-*Increase opportunities for physical activity and sport*
		-*Support healthy schools*
		-*Improve access to healthy food*

In addition to elements specific to the Healthy Living Core Program, BC has implemented other provincial level policies and programs that support chronic disease prevention. Examples are presented in Additional file 2, which are intended to be illustrative rather than exhaustive.

#### Anticipated chronic disease prevention outputs

Interventions aimed towards the adoption of healthy behaviours by British Columbians, primarily related to healthy eating, active living and a smoke-free lifestyle, were a major component of CDP/HL in BC. Some initiatives designed to promote adoption of healthy behaviours were previously and concurrently implemented by the BC Healthy Living Alliance or its member organizations (e.g. the Farm to School Salad Bar and Sip Smart! BC), and evaluation of these initiatives were cited to demonstrate that strategies to promote healthy behaviours were successful [32].

According to BC documents, initiatives that resulted in increased public awareness of chronic diseases, their causes, and ways to prevent them were also seen as essential components of a HL program, i.e., immediate outputs supporting long-term health effects. Tools to increase awareness cited in BC documents included social marketing and public education/awareness campaigns. Increased awareness was seen as building skills-based knowledge, as well as providing the cognitive basis for behaviour change.

Finally, the creation of healthy environments was seen as an important output that is necessary to achieve healthy living and chronic disease prevention. BC’s policy documents reflected a multi-pronged settings approach - attempting to create healthy environments at home, school and work. Some examples include working with partners to create built environments in communities that encourage physical activity, access to the outdoors, access to healthy food and other factors that contribute to chronic disease prevention. It should be noted that the healthy built environment in BC was explicitly addressed in the Healthy Living core program, whereas healthy social environments and settings were addressed in the Healthy Communities core program.

### ON

#### Goals for the chronic disease prevention standard

ON’s overarching goal for CDP, as stated by the Ontario Public Health Standards (OPHS), was “to reduce the burden of preventable chronic diseases of public health importance” which corresponded with the four main categories of disease stated by WHO: cardiovascular diseases, cancer, respiratory diseases and type II diabetes [7], p.18. Some of ON’s explicit goals focussed more on processes such as the process of championing health promotion and driving partnerships for change. To view BC and ON’s goals in more detail, see Table 3.

**Table 3:** Goals for healthy eating and active living strategies, BC and ON

	Goals*
**BC**	• Increased systemic support for healthy living choices, in an integrated manner, at the individual, family, community and regional level.
	• Prevention and reduction of high-risk behaviours, including tobacco use, unhealthy eating and physical inactivity, particularly among young people and vulnerable individuals and groups.
	• Enhanced surveillance, monitoring and evaluation of healthy living trends and interventions. [20], p.10
**ON**	• Champion health promotion in Ontario and inspire individuals, organizations, communities and governments to create a culture of health and well-being.
	• Provide programs, services and incentives that will enhance health and well-being.
	• Make healthy choices easier.
	• Harness the energy and commitment of other Government of Ontario ministries, other levels of government, community partners, the private sector, the media and the public to promote health and well-being for all Ontarians.
	• Make Ontario a leader in health promotion within Canada and internationally [44], p.3

*As no specific goals for healthy living were listed in ON core documents, the authors drew on a supporting document’s goal.

ON highlighted evidence-based policy and practice and drew from a variety of sources to create an evidence base for chronic disease programming. As well, Health Units were expected to review the evidence themselves to guide their planning and evaluation. In the ON Standards, rather than being integrated into the framework as a whole, program planning, evaluation, research and knowledge exchange were included as part of a separate foundational standard that underlies and directs through situational assessments of all programs and services.

ON did not differentiate between an equity lens and a population lens at the framework level, although both equity and a population focus were acknowledged to be important. An equity lens was used for specific interventions but the province did not state equity as an integral component of their public health framework (readers are directed to Pinto, Pauly, Manson, Thanos, Parks & Cox, 2012 for another RePHS comparison paper providing a detailed analysis of BC and ONs’ approach to health equity).

### Components of chronic disease prevention

ON documents, similar to BC, listed four components of healthy eating and active living strategies (see Table 2). Two components were championing public policy and promoting public awareness of parents, caregivers, professionals and the general public. ON also included the community as an integral component, in the context of building healthy community environments. Creating healthy community environments could involve high-level strategies that impacted communities as well as strategies at a local level. ON’s fourth component focused on children and youth, in an effort to improve the environment in which children grow and learn. Other provincial level policies and programs that support chronic disease prevention can be found in Additional file 2.

### Anticipated chronic disease prevention outputs

ON listed several documented initiatives that were aimed at changing population behaviours. A key strategy in prompting a change in attitudes and behaviours in ON documents was social marketing and mass media campaigns; however, there were other complementary strategies in place to encourage adoption of healthy behaviours such as comprehensive program and policy development, e.g., a school food policy that incorporated a skill development component. Another strategy was to ensure that people had the supports they need in order to successfully adopt and maintain healthy behaviours.

ON also placed a strong emphasis on raising awareness - in fact, promoting public awareness and engagement was cited as one of the four key strategies of the former Ontario Ministry of Health Promotion to allow for healthier eating and more active living among Ontarians. As with BC, social marketing and media campaigns/interventions were seen as effective tools for raising awareness. It was also recognized that for messages to be as widespread and effective as possible they needed to be coordinated across a broad range of partners: community organizations, public health units, NGOs, and private sector actors. Public awareness included not only awareness of chronic diseases and CDP/HL options, but also awareness of community health status: risk, protective, and resiliency factors, and the importance of creating healthy environments.

ON documents had a stated vision for use of information as an output by a range of users, perhaps owing to knowledge exchange being positioned as a foundational standard for programs and services. Some knowledge translation and exchange initiatives between policy-makers, practitioners, community members and other partners in CDP programs included using the Healthy Eating and Active Living (HEAL) evaluation logic model in local programming, communicating best practices to staff to build their capacity, and disseminating messages about HEAL to the community through the media. Boards of health were also mandated through the OPHS to disseminate surveillance and population health assessment information to government, public health professionals, other boards of health, and across the larger health system.

Finally, an expected output of CDP strategies in ON documents was the creation of healthier, or more supportive, environments. The term “supportive environment” was defined in a broad sense, encompassing physical, social, and economic dimensions of the surroundings that are necessary for healthy living [28]. It could be a reference to the built environment, but it was also meant in a broad context, in which it is a setting in where activities take place - for example neighbourhoods, schools and workplaces. The creation of healthy environments in this case meant creating supports within those contexts for people to be able to make better choices and live healthier lives.

## Discussion

This study presents a descriptive analysis of public health policies related to healthy eating and active living in BC and ON. Possible explanations for provincial differences were drawn from contextual documents and insights provided by policy makers (GB, JS) on the research team; insights were largely related to their understanding of the motivations underlying the development of and choices made in the policies. Both provinces are in the midst of a transformational public health renewal process that has, according to our analysis, resulted in slightly different approaches to public health policy for chronic disease prevention as it relates to healthy eating and physical activity. These differences and possible explanations for these differences are important to understand for a number of reasons: 1) they serve to contextualize the differences in health outcomes across the two provinces that might eventually be observed; 2) within each province, the explanations might provide insight with respect to the sustainability of local programs and services; and 3) the analysis informs future public health policy directions as the two provinces, and the country, can learn from them. Even if government structures change, the policies that are put in place at a certain point in time will direct intervention efforts, and it is important to keep track of the policies and immediate outputs in order to trace their influence on health outcomes in the years to come [45].

In addition to providing a point of comparison against which subsequent changes in public health policies can be assessed, baseline analysis of reforms being led by ON and BC could be used to inform structural changes that are being spearheaded in other provinces, because public health reform is high on the national agenda. An effective and responsive public health system is an integral part of the larger health care system; likewise an ineffective public health system can increase the stress on other components of the system. This work provides an initial understanding of how reforms might be accomplished through the use of policy instruments that outline direction for public health service delivery. Further, to successfully reduce the burden of chronic disease it is crucial to have sound policies; to date, few studies have examined public health system policies for the prevention of chronic disease. As a first step, this analysis examines what the policies look like, and comments on some anticipated challenges and areas of potential success. This is important because the policy perspective takes a systems look at the future, which can be used to complement a post-hoc look at evaluations of service implementation and delivery.

BC and ONs’ foci on cardiovascular disease, cancers, respiratory illnesses and diabetes were almost the same as the WHO’s recent disease chronic disease priorities in the areas of heart disease and stroke, cancer, asthma and chronic obstructive pulmonary disease, and diabetes (WHO, 2005). However, some aspects of chronic disease prevention policies were unique in structure and content due to distinct program framework organization and philosophical approaches underlying the two frameworks and visions for their implementation. We draw on these contextual factors in the discussion below. Despite the challenges, we and others recognize that, “policies need time to be fully implemented and embedded in practice, before judgements about their impacts on distal outcomes…can be made” [46] p.737. This analysis is an important preliminary step to understanding public health policy implementation.

BC and ON organized the scope and breadth of their CDP/HL core programs differently. Each province’s *unique framework structure* might lead one to conclude erroneously that gaps exist in service provision (a challenge we tried to overcome by using supporting documents along with policy documents). For example, although not organized into a specific component in the Healthy Living core program, the BC Framework did address chronic disease prevention needs that went beyond healthy living through a variety of other core public health programs, for example the “Healthy Infant and Early Child Development (0–6 years),” the Healthy Child and Youth Development”, “Healthy Communities” (which includes a Healthy Schools focus) and “Chronic Disease Prevention” core programs. All programs were inter-related to allow any one core program to build on others and thus cannot be considered in isolation. As another relevant example, the Healthy Living core program might appear to be focussed primarily on individual lifestyle behaviours within the context of the larger framework, but other core programs in a cluster (e.g., the food security and Healthy Communities core programs) in fact focussed on social and environmental supports for chronic disease prevention. The core programs, as well as the public health strategies, lenses, and system capacity elements, need to be thought of as supporting each other within the overall and integrated framework for core public health services within the health care system at large. In contrast, the ON CDP public health standard, in conjunction with the foundational standard, was self-contained to parallel the delivery of services by health units. That is, it could be used as a stand-alone document that was comprehensive in and of itself.

It may well be that the unique organizational and governance structures for public health in the two provinces will be the most important determinants of the longer term impact of the two provinces’ approaches to public health renewal in general, and chronic disease prevention more specifically. In BC, public health is situated within HAs (that are responsible for the totality of health care in the province) and is integrated to a greater or lesser extent in each HA. In keeping with the aim of public health integration into the larger system, BC’s framework explicitly stated that the core public health functions are in fact, functions of the entire HA. This means that public health functions and services may be delivered, not just by traditional public health practitioners or by public health departments, but by others working in the system.

An integrated framework for public health functions, in which each part supports and contributes to the other parts, is congruent with an organizational philosophy of integrated functions and services as in BC. A framework that comprises self-contained and stand-alone standards is more congruent with a public health system in which each standard is delivered through health units acting autonomously from the larger health care system, as in ON. The evidence in support of public health integration into the larger health care system is limited at present, so only time will tell which approach might offer the best advantages for public health service delivery and improved population health outcomes. Our larger study, we hope, will add to our understanding about the benefits or drawbacks of integrated versus autonomous public health systems; this baseline analysis of policies related to chronic disease prevention will contribute to this understanding over the longer term.

The inclusion of *equity* considerations in both policies signifies an important move to mainstream a systematic approach to health and health inequities in public health. The WHO has identified the role of public health in reducing inequities through the priority areas of: social investments; increased accountability and outcomes through stakeholder roles and increased community capacity; inter-sectoral action; knowledge improvement and exchange on how socio-economic factors affect health outcomes; and leadership of public health [47]. BC explicitly specified equity and population lenses for all core programs, highlighting both targeted and universal approaches for health gains, while ON focussed on priority populations from an equity perspective (and did not clearly separate out the two concepts).

ON’s concept of equity draws from the previous Mandatory Health Programs and Services Guidelines for public health, which also had a focus on priority populations. The Mandatory Guidelines were seen as quite prescriptive [23]. Although the focus on priority populations remained, the intent of the new OPHS was to allow health units to practice their independence regarding priority populations since the determination of such groups varies greatly from unit to unit. Which approach – one that explicitly highlights both equity and the needs of specific populations, or one that mostly focuses on priority populations as a proxy for equity [48] – is most effective at improving population health and reducing health inequities for their communities is yet to be seen. Such equity-related health improvements are dependent on changes at the upstream, social determinants of health level, requiring extensive inter-sectoral partnerships and action [45,49]. However, as identified in a policy review of the integration of an equity lens in both BC and ON core PH renewal documents [48], BC had a more explicit commitment to equity in its model core program papers than in ON’s individual public health standards and appears to have resulted in more specific focus on strategies to address health inequities. Follow up on the outcomes of this difference in policy will be important.

Evidence-based decision-making is promoted by governmental and non-governmental bodies worldwide [44,50,51]. Both provinces demonstrated differences in the way that health infrastructure related to information and/or evidence (e.g., training, education, evaluation, knowledge exchange) is conceptualized. The BC Framework (core programs and strategies) was accompanied by the system capacity requirements required for success, such as health information systems and quality management. Also important to understand is that the BC core programs were deliberately supported by a detailed evidence review (or best practices in situations in which research was lacking). Thus, the *role of evidence* is highlighted *in the development* of the BC Framework and the model core program papers (including Healthy Living and Chronic Disease Prevention). In this document, the BC Core Functions Implementation Process included a number of steps toward implementation that built on the integration of evidence into the model core program papers. Responsibility for implementation lies with the HAs, who were responsible for convening a working group to conduct a gap analysis in which current services were examined in relation to the evidence-informed practices described in the model core program papers. As part of this process, some HAs also conducted epidemiological analyses of the current public health issues affecting particular populations within their region and these data were also taken into account in the gap analysis. From this, the working groups in each HA were to develop performance improvement plans to implement the strategies identified in the model core program papers that would address the gaps in service they had identified. Thus, although the CF resource document did not spell out the role of evidence in CF implementation, it was certainly considered. At the same time, many challenges to implementation in BC were identified by those on the ground responsible for implementing core programs.

In ON, the process of OPHS development also involved province-wide consultations, with the result that Research and Knowledge Exchange were positioned as a foundational standard (i.e., a preliminary step in program planning); this standard signalled a need to provide a rationale for decisions where scarce resources had to be applied to complex health issues such as diabetes prevention for a large population. In other words, the role of evidence is highlighted *in the implementation* of the OPHS. This resulted in the need to: develop skills among staff related to data analyses and critical appraisal of literature; increase program planning time; introduce change management with staff regarding skills increase and program changes. This has brought about the awareness that an “evergreen” process is required where new data and literature are incorporated into revised standards using a process that includes academics, field/practitioner experts and Ministry staff, as was carried out in the BC example. While ON Health Units are expected to locate, interpret and synthesize evidence for programs, which is a well-documented challenge, they are supported in their capacity to do this by Public Health Ontario, a crown corporation dedicated to providing expert scientific and technical advice around public health issues, as well as professional development services, representing a shared responsibility for evidence-based programming [52].

*Unique policy formulation approaches* motivated the development and intent of the new public health functions/standards, adding another layer of complexity to understanding the policies in the two provinces. Historically, BC did not have a previous public health framework from which to build. BC’s approach to developing Core Functions was very consultative, and high-level guidance was provided at the provincial level but details were left to the HAs. The intent was for HAs to address the core public health programs in an integrated way within the overarching framework. Further, the CF framework assumed that it is not just traditional public health practitioners that would be doing “public health” programming. The notion of collaboration within the health sector and between sectors was built into the assumptions of the framework. As well, there exist other community structures, organizations or partnerships that support or have supported Healthy Living and Chronic Disease Prevention programs, e.g., the BC Healthy Living Alliance, and ActNow BC.

In ON, the Foundational standard was designed to identify the steps in public health program or policy development. For example, Step 1 would include gathering data on the health issue; Step 2 would involve reviewing literature on interventions; then in Step 3 staff would determine the most effective strategy given the problem and identify interventions either proven to be effective or, in many cases, interventions that show promise; Step 4 would involve evidence-informed decision making to confirm if staff and resources are available (if not, train staff, obtain resources through grants, partnerships, etc.); Step 5 related to evaluation and knowledge exchange – publish, present, etc. It is in these steps that issues such as equity, determinants of health, etc., were meant to be given strong consideration in the context of all other influencing factors on the health problem – and in the context of how to effectively intervene. Although ON has legislated requirements around the OPHS, these new standards were intended to empower staff to assess local health status and to analyze more data and literature when designing a program that is effective locally. The Foundational standard represents a significant shift from previous Mandatory Health Programs and Services Guidelines that were prescriptive, but it still represents a revision of a previous policy. This contrast in policy development – innovative policy reform in BC and policy renewal with incremental changes in ON – might be an important difference when assessing future substantive and procedural successes and failures with respect to implementation and outcomes.

### Next steps

This work leads to obvious next steps that include: assessing policy implementation through embeddedness in practice, measuring anticipated outputs and then measuring long-term outcomes related to these public health policies. Outcomes have been estimated to surface between 5 – 25 years after intervention implementation [45], suggesting that both short and immediate term definitions of ‘outcomes’ (not to mention population-level impacts) ought to be given due consideration. Our larger research program aims to understand the implementation of the policies against the backdrop of the policy content described in this paper. This is important because it reveals whether less-than-expected outcomes are due to the policy itself or failed implementation efforts [53].

Findings from this work ought to be considered in light of certain limitations. The scope of our study was limited to policy documents at the provincial level. A different search strategy could have uncovered other documents at the HA or Health Unit level that might have provided further insights about priorities, expected outcomes, etc. Additionally, because this analysis focused on traditional elements of the public health system - primarily government ministries, HAs, and public health units, it did not allow for inclusion of many other civil society actors that form integral components of the public health system - such as public health agencies and organizations, professional associations, non-profit organizations, and community organizations. While as a result the analysis cannot provide a comprehensive picture of all public health activities, its aim was to compare policies (and subsequent programs) of provincial public health leadership. In subsequent papers, as the RePHS project progresses, it will be possible to make much more confident inferences about how different policy settings affect program implementation within these formal public health structures. However, in order to examine health outcomes at the community level, other actors and systems will have to be taken into account.

As well, our analysis was limited to one influence (policy documents) among many in the policy-making process, such as resources, value and power. Nevertheless, these limitations are compensated by the systematic process of collecting and analysing documents in this study. As well, the authorship team comes with varied expertise – academics and decision-makers from both provinces – which brings a more balanced interpretation of findings than might have been presented by a traditional scientist-only research team. This balanced interpretation represents a unique strength of this work.

## Conclusion

Ratzan notes that, “The noncommunicable disease threat will require innovative responses and concerted action – both public and private – to reverse the troubling trends and turn the tide toward health and well being” [54], p.2. This analysis describes the different recent approaches in BC and ON to addressing risk factors related to chronic diseases. What becomes clear is that the two provincial policies present different approaches to support the implementation of related programs. While ON initiated the process using historical public health guidelines as a jumping off point, engaging in incremental policy change, BC created an entirely new CF Framework, engaging in policy change that is somewhat experimental. In BC the HAs clustered core programs at the level of service delivery (i.e., in anticipation of synergistic health gains in chronic disease prevention) based on analysis of regional health inequities and gaps in service. ON encouraged health units to develop rationales, local evidence reviews and local health standards to support health improvements. The description of the two policies, and the contextual explanations for the differences, are important because the analysis permits a comparison of reforms associated with different public health structures (regional HAs and public health units), which are common structures for the delivery of public health services in other countries.

## Abbreviations

BC: British Columbia; CVD: Cardiovascular disease; CF Framework: Core functions of public health framework; CVP: Chronic disease prevention; HA: Health authority; HEAL: Healthy eating and active living; HL: Healthy living; MOH: Medical officers of health; MOHLTC: Ontario Ministry of Health and Long-term Care; NGO: Non-governmental organization; ON: Ontario; OPHS: Ontario public health standards; PHAC: Public health agency of Canada; RePHS: Renewal of public health systems; WHO: World health organization.

## Competing interests

The authors declare that they have no competing interests. The views expressed in this article are those of the authors and do not necessarily represent the official position of their organizations.

## Authors’ contributions

AK led the conceptualization of this project, developed the preliminary codebook, helped to revise and finalize the codebook, participated in the document analysis, helped to write the first draft of the manuscript, and led the process of revising the manuscript to address reviewers’ feedback. DG helped to revise and finalize the codebook, participated in the document analysis and helped to write the first and revised drafts of the manuscript. MM (with other principal investigators) conceptualized the larger study of which this is a part and wrote the funding proposal, participated in conceptualizing this project, helped to revise and finalize the codebook, participated in the document analysis, and provided significant feedback and editing on the manuscript. DA helped to revise and finalize the codebook, participated in the document analysis and provided feedback on the manuscript. GB participated in the document analysis, provided key contextual knowledge and feedback on the manuscript. JS participated in the document analysis, provided key contextual knowledge and feedback on the manuscript. All authors read and approved the final manuscript.

## Pre-publication history

The pre-publication history for this paper can be accessed here:

http://www.biomedcentral.com/1471-2458/13/934/prepub

## Supplementary Material

Additional file 1 **High-level summary of the CDP/HL landscape.** A summary of the federal and provincial context surrounding chronic disease prevention and healthy living policy in Ontario and British Columbia, including relevant legislation, policies, organizations and structures. Click here for file

Additional file 2 **Examples of programs that support the components of HL/CDP policies in BC and ON.** Select programs in each province that fall under each component of healthy living/chronic disease prevention policies in Ontario and British Columbia. Click here for file
